# Digital Inclusion Driving the Silver Economy among Older Adults in China: A Supply-Demand Theoretical Perspective

**DOI:** 10.1093/geroni/igaf122.447

**Published:** 2025-12-31

**Authors:** Chuanzheng Xu, Jing Ye

**Affiliations:** Wuhan University, Wuhan, Hubei, China (People’s Republic); Wuhan University, Wuhan, Hubei, China (People’s Republic)

## Abstract

**Background:**

Amid accelerating global aging, the digital divide poses systemic threats to social equity. This study applies a supply-demand theoretical framework to analyze how digital inclusion bridges supply-demand gaps in the silver economy, examining dual mechanisms of supply optimization and consumption stimulation among older adults.

**Methodology and Data:**

Using Chinese provincial panel data (2012-2022) and the China Health and Retirement Longitudinal Study (2013, 2015, 2018, 2020), this research constructs a tripartite digital inclusion index (accessibility-utilization-benefit) using the entropy weight method as the explanatory variable. To assess its causal effects on the silver economy’s supply-demand dynamics, this study integrates supply-side industrial evolution with demand-side consumption trajectories as outcome variables.

**Findings:**

(1) Digital inclusion significantly drives silver economy growth, expanding aging-related industry scale and older adults’ consumption. (2) The regional business environment plays a mediating role between digital inclusion and the supply-demand expansion in the silver economy. (3) Less-developed regions demonstrate a “catch-up effect” resulting from digital inclusion improvements.

**Discussion:**

To unlock older adults’ economic potential and address aging challenges equitably, digital inclusion strategies must integrate technological empowerment with institutional reforms. This requires expanding digital infrastructure in underserved regions and optimizing the business environment to foster inclusive growth in the silver economy.

